# Three-Year Outcomes of Type 2 Diabetes Remission Following Sleeve Gastrectomy and Roux-en-Y Gastric Bypass in Patients With BMI ≥35: A Single-Center Cohort Study

**DOI:** 10.7759/cureus.94578

**Published:** 2025-10-14

**Authors:** Muhammad U Khan, Amer Andrabi, Nouran Alkhoori, Javed A Raza

**Affiliations:** 1 General Surgery, University Hospitals Birmingham, Birmingham, GBR; 2 General Surgery, Cleveland Clinic Abu Dhabi, Abu Dhabi, ARE

**Keywords:** bariatric surgery, diabetes remission, obesity, roux-en-y gastric bypass, sleeve gastrectomy, type 2 diabetes mellitus

## Abstract

Background

Bariatric surgery is an effective treatment for obesity and type 2 diabetes mellitus (T2DM). Sleeve Gastrectomy (SG) and Roux-en-Y Gastric Bypass (RYGB) are the two most common procedures, but their outcomes in Middle Eastern populations are less well studied.

Objective

To describe and compare the three-year outcomes of T2DM remission after SG and RYGB in patients with BMI ≥35.

Methods

This was a retrospective cohort study of 162 patients with T2DM who underwent laparoscopic SG (n=84) or RYGB (n=78) at the Cleveland Clinic Abu Dhabi between 2017 and 2018. Patients were followed for at least three years. The main outcome was diabetes remission, defined as HbA1c <6.5% without glucose-lowering medication. We also examined changes in medication use and differences between males and females.

Results

The age range of patients was 39-74 years for the SG group and 41-72 years for the RYGB group, with no significant difference between them. At three years, 39.3% of patients who had SG and 52.6% of those who had RYGB achieved remission. Among patients on insulin at baseline, 30.0% (SG) and 37.9% (RYGB) went into remission. About half of the insulin users in each group were able to switch to oral medications only, and fewer remained on insulin after RYGB (13.8%) compared with SG (20.0%). In patients using only oral agents before surgery, remission was more frequent after RYGB (61.2%) than SG (44.4%). Remission rates were similar between men and women, indicating that gender did not significantly influence outcomes.

Conclusion

Both SG and RYGB were associated with remission of T2DM at three years in patients with BMI ≥35, with a higher proportion of remission observed after RYGB. Gender did not appear to affect outcomes.

## Introduction

Obesity and type 2 diabetes mellitus (T2DM) are major global health challenges, with particularly high prevalence in the Middle East due to lifestyle and dietary transitions. The burden of these conditions places increasing strain on healthcare systems in the region.

Bariatric surgery has emerged as a highly effective treatment for both weight loss and T2DM remission. Sleeve Gastrectomy (SG) and Roux-en-Y gastric bypass (RYGB) are the two most common procedures worldwide. SG primarily reduces gastric capacity and suppresses ghrelin secretion, whereas RYGB induces additional metabolic changes such as increased secretion of glucagon-like peptide-1 (GLP-1) and peptide YY (PYY), altered bile acid signaling, and enhanced insulin sensitivity.

Randomized controlled trials and long-term cohort studies demonstrate the superiority of bariatric surgery over medical therapy. The Systemic Therapy for Advancing or Metastatic Prostate Cancer: Evaluation of Drug Efficacy (STAMPEDE) trial confirmed durable benefits of surgery over intensive medical management, with RYGB often outperforming SG [1,2]. The Alliance of Randomized Trials of Medicine vs. Metabolic Surgery in Type 2 Diabetes (ARMMS-T2D) consortium reinforced the long-term metabolic benefits of surgery [3]. The Sleeve vs Bypass (SLEEVEPASS) trial provided 10-year comparative outcomes of SG and RYGB [4]. Mingrone et al. also demonstrated greater remission with surgery than medical therapy [5]. Reviews and meta-analyses further highlight these findings [6,7].

Despite extensive data from Western countries, evidence from Middle Eastern populations is limited. This study evaluated three-year T2DM remission after SG and RYGB in patients with BMI ≥35 at a tertiary academic hospital in the United Arab Emirates.

## Materials and methods

Study design and setting

This retrospective, single-center cohort study was conducted at the Cleveland Clinic Abu Dhabi, UAE. Surgeries were performed between January 2017 and December 2018, with a follow-up period of at least three years.

Eligibility criteria

The selection of SG versus RYGB was individualized, based on multidisciplinary team discussion, patient preference, comorbidity profile, and surgeon judgment in accordance with institutional metabolic surgery protocols.

Inclusion criteria included adults (≥18 years), BMI ≥35, diagnosis of T2DM, having undergone primary SG or RYGB, and having at least three years of follow-up data. Exclusion criteria involved adults with type 1 diabetes, prior bariatric or foregut surgery, revision procedures, or incomplete follow-up.

Data collection

Demographics, including age range and sex distribution, baseline diabetes therapy (oral vs insulin), and surgical procedure, were extracted from hospital records. Outcomes included remission status, medication transitions, and gender-stratified remission at three years.

Definitions

Complete remission: HbA1c <6.5% without glucose-lowering medication for a minimum of three months (per American Diabetes Association (ADA) standards [8]).

Persistent insulin use: patients who remained on insulin therapy at the three-year follow-up visit.

Statistical analysis

Continuous variables are reported as mean ± standard deviation (SD) or range. Categorical variables are expressed as n (%). Group comparisons used chi-square or Fisher’s exact tests for categorical data, and t-tests or Mann-Whitney U tests for continuous data. A two-sided p-value <0.05 was considered statistically significant.

## Results

Baseline characteristics

A total of 162 patients were included in the study, with 84 undergoing SG and 78 undergoing RYGB. The age distribution was broadly similar between the two groups (SG: 39-74 years; RYGB: 41-72 years). The SG cohort consisted of 38 (45.2%) male patients and 46 (54.8%) female patients, while the RYGB cohort comprised 46 (59.0%) male patients and 32 (41.0%) female patients. Approximately one-third of patients in both groups were on insulin therapy at baseline (30/84, 35.7% in SG; 29/78, 37.2% in RYGB), and the remainder were managed with oral agents. These baseline data demonstrate comparable demographic and treatment profiles between SG and RYGB, suggesting that the groups were clinically well-matched for subsequent comparisons (Table 1).

**Table 1:** Baseline characteristics of both study groups SG: Sleeve Gastrectomy; RYGB: Roux-en-Y Gastric Bypass.

Variable	SG (n=84)	RYGB (n=78)
Age range, years	39-74	41-72
Male, n (%)	38 (45.2)	46 (59.0)
Female, n (%)	46 (54.8)	32 (41.0)
Insulin users, n (%)	30 (35.7)	29 (37.2)
Oral-agent users, n (%)	54 (64.3)	49 (62.8)

Primary outcome: diabetes remission at three years

At the three-year follow-up, 33 (39.3%) of SG patients achieved complete diabetes remission compared with 41 (52.6%)of RYGB patients. Although the absolute remission difference of 13.3% favored RYGB, this was not statistically significant (p=0.12). This suggests that while both procedures were effective in inducing remission, RYGB may confer an additional, though not statistically proven, advantage in this cohort (Table 2).

**Table 2:** Three-year diabetes outcomes in patients undergoing SG vs RYGB Values are compared between sleeve gastrectomy (SG) and Roux-en-Y Gastric Bypass (RYGB) using Pearson chi-square tests with Yates continuity correction (2×2); two-sided p-values.

Outcome	SG n/N (%)	RYGB n/N (%)	Test statistic (χ²)	p-value
Overall remission	33/84 (39.3)	41/78 (52.6)	2.36	0.12
Baseline insulin users – Remission	9/30 (30.0)	11/29 (37.9)	0.14	0.71
Baseline insulin users – Insulin → oral	15/30 (50.0)	14/29 (48.3)	0	1
Baseline insulin users – Persistent insulin	6/30 (20.0)	4/29 (13.8)	0.08	0.77
Baseline oral-agent users – Remission	24/54 (44.4)	30/49 (61.2)	2.27	0.13

Secondary outcomes: subgroup analysis by baseline therapy

Among patients on insulin at baseline, remission at the three-year follow-up was achieved in 9/30 (30.0%) SG and 11/29 (37.9%) RYGB patients (p=0.71). Importantly, by three years, half of the insulin users in both groups were able to discontinue insulin and continue on oral therapy alone (SG: 15/30, 50.0%; RYGB: 14/29, 48.3%). Persistent insulin use at three years was less common after RYGB (4/29, 13.8%) compared with SG (6/30, 20.0%), though this difference was not statistically significant (p=0.77).

In patients using only oral agents at baseline, remission was achieved in 24/54 (44.4%) after SG and 30/49 (61.2%) after RYGB (p=0.13). Although not statistically significant, these results again suggest a potential benefit of RYGB in facilitating remission, particularly in patients with less advanced diabetes at baseline.

Gender-stratified outcomes

Gender did not significantly affect remission outcomes. Within SG, remission was nearly identical between male patients (15/38, 39.5%) and female patients (18/46, 39.1%; p=0.85). Similarly, in RYGB, male patients (24/46, 52.2%) and female patients (17/32, 53.1%; p=0.88) achieved remission at comparable rates. This indicates that the type of procedure and baseline diabetes severity may play a larger role than gender in influencing remission outcomes (Table 3).

**Table 3:** Gender-stratified three-year remission rates SG: sleeve gastrectomy; RYGB: Roux-en-Y Gastric Bypass. Within each procedure (SG or RYGB), remission proportions among male and female patients were compared using Pearson chi-square tests with Yates continuity correction (2×2); two-sided p-values. The same χ² and p apply to the male-female patient pair within a procedure, hence they repeat across the two rows.

Group	Remission, n/N (%)	Test statistic (χ²)	p-value (male vs female)
Male patients undergoing SG (n=38)	15/38 (39.5)	0	1
Female patients undergoing SG (n=46)	18/46 (39.1)	0	1
Male patients undergoing RYGB (n=46)	24/46 (52.2)	0	1
Female patients undergoing RYGB (n=32)	17/32 (53.1)	0	1

Summary of findings

Overall, RYGB patients showed numerically higher remission rates, particularly among those on oral therapy at baseline, and fewer remained insulin-dependent at three years compared with SG. However, statistical significance was not reached, likely due to limited sample size. These findings highlighted both procedures as effective metabolic interventions, while suggesting a possible incremental advantage of RYGB that warrants further investigation in larger regional cohorts.

## Discussion

This study adds valuable evidence from a Middle Eastern cohort, demonstrating that both SG and RYGB lead to significant T2DM improvements at three years in patients with BMI ≥35. Although the remission was numerically higher after RYGB (52.6%) compared to SG (39.3%), the difference did not achieve statistical significance. Nonetheless, the trend aligns with global data suggesting that RYGB may provide superior metabolic benefits compared with SG.

Comparison with international literature

Our remission rates were consistent with prior landmark trials. The STAMPEDE trial showed that RYGB produced greater and more sustained diabetes remission compared with SG and intensive medical therapy, with durable effects at five years [1,2]. Similarly, the ARMMS-T2D consortium reported persistent benefits of bariatric surgery at five years, reinforcing surgery as superior to medical therapy [3]. The SLEEVEPASS trial demonstrated durable long-term remission for both SG and RYGB at 10 years, though RYGB consistently showed a modest advantage [4].

Mingrone et al. also observed significantly higher remission with surgery compared to medical therapy in a randomized trial [5]. Meta-analyses have confirmed these patterns, showing RYGB achieving higher rates of diabetes remission, reduced need for medication, and improved insulin sensitivity compared with SG [7-9]. Our findings are congruent with this body of evidence, despite statistical non-significance, likely reflecting the limited sample size rather than a lack of true effect.

Mechanistic insights

The physiological differences between SG and RYGB provide a plausible explanation for observed outcomes. RYGB enhances glucagon-like peptide-1 (GLP-1) and peptide YY (PYY) secretion, alters bile acid signaling, and promotes insulin sensitivity, leading to rapid glycemic improvements independent of weight loss [6]. SG also improves glycemic control, primarily through gastric restriction and reduced ghrelin levels, but may have weaker hormonal and metabolic effects compared with RYGB. These mechanisms support the consistently higher remission rates seen with RYGB.

Role of baseline therapy

Subgroup analysis suggested that patients treated with oral agents at baseline had higher remission rates compared with insulin users, particularly after RYGB (61.2% vs 37.9%). This aligns with prior reports that earlier surgical intervention, before progression to insulin dependence, is associated with greater likelihood of remission [10,11]. These findings underscore the importance of timely surgical referral.

Gender and outcomes

Our study found no significant effect of gender on remission outcomes, consistent with other literature indicating sex is not a major determinant of diabetes remission after bariatric surgery [10]. This suggests that metabolic effects of surgery transcend biological sex and that procedure type and baseline disease severity are more relevant predictors.

Strengths and limitations

Strengths of the study included complete three-year follow-up and contribution of regional data from a Middle Eastern cohort, where published evidence is sparse. Limitations include the retrospective design, single-center scope, relatively small sample size limiting power to detect statistical significance, and absence of detailed baseline glycemic parameters such as HbA1c levels and duration of diabetes. Additionally, we did not report on weight loss trajectories, which may have provided additional context for remission outcomes.

Detailed insulin dosage data were not uniformly available in the retrospective dataset, which limited the analysis of percentage dose reduction among patients remaining on insulin.

Clinical implications

Despite limitations, this study reinforces the metabolic efficacy of both SG and RYGB, while suggesting potential incremental benefits with RYGB. Clinicians should consider RYGB for patients prioritizing glycemic control, particularly those still on oral therapy. However, SG remains an effective option, especially where lower surgical risk or anatomical considerations favor a restrictive procedure. These findings can aid shared decision-making between patients and surgical teams in the Middle Eastern context. Nevertheless, given the retrospective design and limited sample size, these observations should be interpreted with caution, as the study was not powered to detect small but potentially clinically relevant differences between procedures.

Our findings also align with the Longitudinal Assessment of Bariatric Surgery (LABS) study, which reported sustained weight loss and metabolic benefits at three years following bariatric surgery, reinforcing the durability of surgical outcomes [12].

## Conclusions

In this study of patients with obesity and type 2 diabetes, both SG and RYGB resulted in a substantial metabolic improvement after three years. A greater proportion of patients who underwent RYGB achieved diabetes remission, although both operations were effective in improving glycemic control and reducing the need for medication. The type of surgery, rather than gender, appeared to have a greater impact on long-term metabolic outcomes.

These findings highlight the importance of individualized surgical selection based on the patient’s metabolic goals, comorbidities, and overall risk profile. Earlier surgical intervention before progression to advanced diabetes may increase the likelihood of remission. Further multicenter studies with larger sample sizes and longer follow-up are encouraged to validate and expand on these results in regional populations.
